# Síndrome da encefalopatia posterior reversível em paciente com COVID-19 submetida à oxigenação por membrana extracorpórea

**DOI:** 10.5935/0103-507X.20210067

**Published:** 2021

**Authors:** João Francisco Martins, Luis Rito Cruz, Daniela Jardim Pereira, Jose Eduardo Sousa, Paulo Martins

**Affiliations:** 1 Serviço de Medicina Intensiva, Centro Hospitalar e Universitário de Coimbra - Coimbra, Portugal.; 2 Serviço de Imagem Médica, Centro Hospitalar e Universitário de Coimbra - Coimbra, Portugal.

**Keywords:** Manifestações neurológicas, Encefalopatias, Infecções por coronavírus, COVID-19, Síndrome do desconforto respiratório agudo, Oxigenação por membrana extracorpórea

## Abstract

Uma mulher com 63 anos de idade compareceu ao pronto-socorro com história aguda de febre, prostração e dispneia. Recebeu diagnóstico de quadro grave da COVID-19 e síndrome do desconforto respiratório agudo. Apesar de suporte clínico intensivo, cumpriu os critérios para ser submetida à oxigenação venovenosa por membrana extracorpórea. No dia 34, após 7 dias de desmame da sedação com evolução positiva de seu quadro neurológico, apresentou uma crise tônico-clônica generalizada limitada, não relacionada com desequilíbrio hidroeletrolítico ou metabólico, que levou à necessidade de investigação diagnóstica. Seus exames de imagem cerebral revelaram síndrome da encefalopatia posterior reversível. Este caso enfatiza a questão das complicações neurológicas em pacientes com COVID-19 grave e a importância do diagnóstico e suporte precoces.

## INTRODUCTION

Coronavirus disease 2019 (COVID-19) is a rapidly evolving pandemic that emerged in Wuhan, China, and so far, no specific treatment has been considered effective except supportive care.^(1)^ The Extracorporeal Life Support Organization (ELSO) guidelines for extracorporeal membrane oxygenation (ECMO) in COVID-19 patients recommend administering veno-venous extracorporeal membrane oxygenation (VV-ECMO) for the treatment of COVID-19-related acute respiratory distress syndrome (ARDS) in expert centers.^(2)^

Neurologic complications are becoming increasingly recognized in patients with severe COVID-19 infections.^(3)^ The most common neurologic symptoms include headache, anosmia, and ageusia. Other findings include stroke, impairment of consciousness, coma, seizure and encephalopathy.^(4)^ Damage within the central nervous system might be caused directly by the virus or by the body’s innate and adaptive immune responses to infection. We describe one patient with severe COVID-19 ARDS who underwent VV-ECMO in whom brain imaging showed posterior reversible encephalopathy syndrome (PRES).

## CASE REPORT

A 63-year-old woman with a past medical history of obesity and arterial hypertension presented to the emergency department of a secondary hospital with a 7-day history of fever, dry cough, hyposmia and myalgia. At admission, she was alert with a respiratory rate of 22 cycles per minute, pulse rate of 90 beats per minute, and oxygen saturation of 90%. Laboratory tests revealed lymphopenia (1.55 x 10^9^) and elevated C-reactive protein (10.62mg/dL). COVID-19 testing was performed by nasopharyngeal and throat swabs and was positive for severe acute respiratory syndrome coronavirus 2 (SARS-CoV-2) by polymerase chain reaction.

The situation rapidly evolved, with fever, prostration and worsening dyspnea. Arterial blood gas analysis showed severe respiratory failure, and chest X-ray evidenced diffuse bilateral pulmonary infiltrates. She was intubated and mechanically ventilated, but her respiratory status continued to deteriorate despite optimized critical care, including prone positioning ventilation and neuromuscular blockade. Her chest computed tomography scan after intubation revealed extensive multifocal ground-glass opacities bilaterally, without any pulmonary embolism. The initial ventilator settings were volume-controlled ventilation with a tidal volume of 360mL (6mL/kg of ideal body weight), respiratory rate of 14 breaths per minute, positive end-expiratory pressure (PEEP) of 14 cmH_2_O and static compliance 44cmH_2_O.

On the second day of admission, arterial blood gas revealed a pH of 7.34, partial pressure of carbon dioxide (PaCO_2_) of 53mmHg, partial pressure of oxygen (PaO_2_) of 102mmHg and bicarbonate (HCO_3_) of 29.7mmol/L, with a partial pressure of oxygen/fraction of inspired oxygen (PaO_2_/FiO_2_) ratio of 98. She met the indications for VV-ECMO and was transferred to our intensive care unit (ICU), which has an established VV-ECMO program. The procedure was performed safely, and no complications occurred. Arterial blood gas analysis 3 hours after beginning VV-ECMO showed a pH of 7.386, PaCO_2_ of 43mmHg, PaO_2_ of 94.3mmHg, and HCO_3_of 25.4mmol/L, with no abrupt variation in PaCO_2_. Early nutrition and rehabilitation were begun after VV-ECMO introduction.

On day 14 of admission, the patient developed ventilator-associated pneumonia due to *Pseudomonas aeruginosa* and had completed 7 days of antibiotic therapy with ceftazidime and vancomycin with a favorable response. On day 20 of admission, her chest radiography and lung compliance improved, and she was successfully decannulated after 455 hours of VV-ECMO support and was tracheostomized 7 days later. The patient maintained good renal function throughout her hospitalization.

On day 34 of admission, after 7 days of sedation weaning with a positive evolution of her neurologic status, a limited generalized tonic-clonic seizure led to a diagnostic investigation.

### Investigation

Blood tests revealed decreasing C-reactive protein (6.11mg/dL), procalcitonin (0.07ng/mL), fibrinogen (307ng/mL) and ferritin levels (2,802ng/mL) but increasing levels of D-dimer (18,021ng/mL) and interleukin 6 (10.4pg/mL) on the day before the development of symptoms. None of the drugs administered to the patient were associated with PRES, namely, immunosuppressive, immunomodulating and chemotherapeutic drugs.

**Figure 1:**
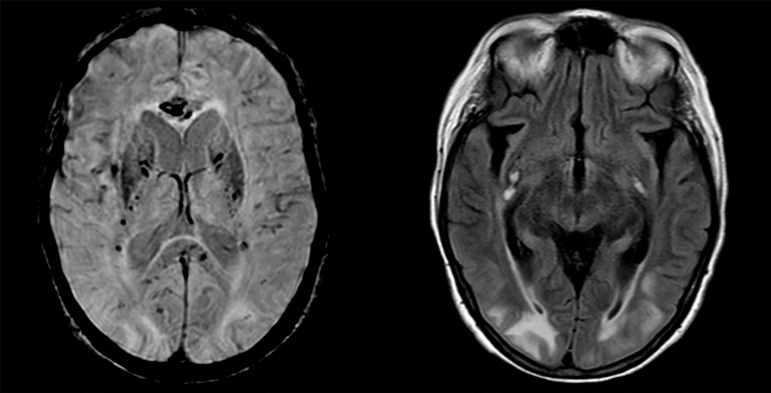
Axial susceptibility-weighted images demonstrate numerous punctate microhemorrhagic foci within the deep and subcortical white matter, particularly in the internal capsule and within the corpus callosum. Fluid-attenuated inversion recovery images show symmetric and confluent hyperintensity affecting the juxtacortical and subcortical white matter, primarily affecting occipital and parietal regions. There were three infracentimetric subacute hemorrhages in the external capsules, with fluid-attenuated inversion recovery and T1 hyperintensity.

A brain magnetic resonance imaging (MRI) scan was performed, and fluid-attenuated inversion recovery (FLAIR) images demonstrated symmetric and confluent hyperintensity affecting the juxtacortical and subcortical white matter, affecting primarily her occipital and parietal regions but also her frontal, temporal and left cerebellar hemispheres. The deep gray nuclei were spared, and no area of restricted diffusion or contrast enhancement was present. Susceptibility-weighted imaging showed multiple punctate microhemorrhages affecting superficial and deep white matter but relatively sparing the area of FLAIR hyperintensity. Punctate microhemorrhages predominantly involved the juxtacortical white matter and the corpus callosum (especially the genu and splenium). Additionally, there were three infracentimetric subacute hemorrhages in the external capsules, with T1 hyperintensity (Figure 1).

The cerebrospinal fluid specimen was negative for SARS-CoV-2 and showed protein (31mg/dL), glucose (74mg/dL) and lactate (1.89mmol/L) within normal limits.

### Outcome

She had nearly recovered from the neurologic deficits despite critical-illness myopathy and was discharged after 44 days in the hospital to a secondary hospital’s subacute rehabilitation facility, having nearly returned to her baseline mentation.

## DISCUSSION

According to the interim guidance formulated by ELSO, ECMO should be considered a rescue therapy for COVID-19 with refractory hypoxemia and worsening hypercapnia despite optimized traditional therapies, in particular lung-protective ventilation, prone positioning and high PEEP.^(2)^ Extracorporeal membrane oxygenation is being used as rescue therapy in patients with severe lung injury secondary to COVID-19, but it is associated with several complications, such as neurologic injuries, that can be a significant cause of morbidity and mortality. Although the most frequent neurological complications related to ECMO are intracerebral hemorrhage and ischemic stroke,^(5)^ to the best of our knowledge, this is the first report of patients with COVID-19 supported with ECMO who presented PRES.

In fact, the described MRI findings are highly suggestive of PRES and indicate dysfunction of the brain’s vascular autoregulation and endothelial dysfunction. Although the patient also had a prolonged intubation period with fluctuating blood pressures, namely, mean arterial pressures between 95 and 130mmHg, endothelial dysfunction secondary to COVID-19 could also have contributed to the PRES. Cerebral blood flow and autoregulation can also be affected during VV-ECMO, including abrupt PaO2 and PaCO2 changes on ECMO initiation.^(6)^ The presence of SARS-CoV-2 in slow-flowing cerebral microcirculation may facilitate the interaction of the virus spike protein with endothelial ACE2 receptors, initiating a cycle of viral budding, impairing autoregulation and increasing the risk of capillary rupture.^(7)^ Accordingly, white matter microhemorrhages might reinforce this process or, alternatively, might also be related to hypoxia, which is common in ECMO-treated patients, and translate into posthypoxic leukoencephalopathy.

## CONCLUSION

Our patient recovered with medical management and nursing rehabilitation, in line with the favorable clinical results described in most cases of non-COVID-19 posterior reversible encephalopathy syndrome.
